# Structural phase transition, equation of state and phase diagram of functional rare earth sesquioxide ceramics (Eu_1−*x*_La_*x*_)_2_O_3_

**DOI:** 10.1038/s41598-020-68400-9

**Published:** 2020-07-16

**Authors:** K. A. Irshad, V. Srihari, S. Kalavathi, N. V. Chandra Shekar

**Affiliations:** 1 High Pressure Physics Section, Condensed Matter Physics Division, Materials Science Group, HBNI, Indira Gandhi Centre for Atomic Research, Kalpakkam, 603102 India; 2 High Pressure and Synchrotron Radiation Physics Division, Bhabha Atomic Research Centre, Mumbai, 400085 India

**Keywords:** Phase transitions and critical phenomena, Structure of solids and liquids, Structure of solids and liquids, Phase transitions and critical phenomena, Thermodynamics, Chemical physics

## Abstract

The intriguing functional nature of ceramics containing rare earth sesquioxide (RES) is associated with the type of polymorphic structure they crystallize into. They prefer to be in the cubic, monoclinic or hexagonal structure in the increasing order of cation size, R_RE_. Since the functional properties of these ceramics varies with R_RE_, temperature and pressure, a systematic investigation delineating the cation size effect is indispensable. In the present work we report the structural stability and compressibility behaviour of the RES ceramics, (Eu_1−*x*_La_*x*_)_2_O_3_, of RESs with dissimilar structure and significant difference in cationic radii. The selected compositions of (Eu_1−*x*_La_*x*_)_2_O_3_ have been studied using the *in-situ* high pressure synchrotron X-ray diffraction and the structural parameters obtained through Rietveld refinement. The cubic structure, which is stable for 0.95 Å $\le$ R_RE_ $<\,0.98$ Å at ambient temperature and pressure (ATP), prefers a cubic to hexagonal transition at high pressures. The biphasic region of cubic and monoclinic structure, which is stable for 0.98 Å $\le$ R_RE_ $<\,1.025$ Å at ATP, prefers a cubic/monoclinic to hexagonal transition at high pressures. Further, in the biphasic region of monoclinic and hexagonal structure, observed for 1.025 Å $\le$R_RE_ $<\,1.055$ Å, the monoclinic phase is found to be progressing towards the hexagonal phase with increasing pressure. The pure hexagonal phase obtained for 1.055 Å $\le$ R_RE_ $\le$ 1.10 Å is found to be structurally stable at high pressures. The bulk moduli are obtained from the Birch–Murnaghan equation of state fit to the compressibility data and its dependance on the cation size is discussed. The microstrain induced by the difference in cation size causes an internal pressure in the crystal structure leading to a reduction in the bulk modulus of $\textit{x}=0.2$ and 0.6. A pressure–concentration (P–*x*) phase diagram upto a pressure of 25 GPa is constructed for (Eu_1−*x*_La_*x*_)_2_O_3_. This would provide an insight to the fundamental and technological aspects of these materials and the RESs in general.

## Introduction

Rare earth sesquioxides (RESs) are promising candidate materials for a wide variety of applications in many of the technologically important fields. They are useful candidates as scintillating materials, phosphor materials, wave guides, superconducting materials and many others^1–4^. These oxides are known to exist in three different polymorphic structures at ambient conditions. They are designated as C-type (cubic), B-type (monoclinic) and A-type (hexagonal) structure^5,6^. The C-type structure belongs to the cubic crystal system crystallizing in the *Ia*$\bar{3}$ space group (SG). The rare earth (RE) cations in this structure occupy two different Wyckoff’s site 8*b* and 24*d* and are octahedrally co-ordinated with the oxygen atoms. The B-type structure with SG *C*2/*m* is shown by the medium cation sized RES (Sm_2_O_3_, Eu_2_O_3_ and Gd_2_O_3_) and the cations in this structure occupy four 4*i* Wyckoff’s sites and the oxygen occupies the three 4*i* Wyckoff’s sites. Here, the cations are in six and seven fold co-ordination with the oxygen atoms. The RES with A-type structure crystallises in the SG *P*$\bar{3}$*m*1 with the cations occupying at 2*d* Wyckoff’s site and anion occupying at 2*d* and 1*a* Wyckoff’s sites. RE cations over here are in seven fold co-ordinated with the oxygen atoms. As the crystal structure of these oxides is mainly dependent on the cation size, the solid solutions of them are extensively studied to induce or to improve certain physical or chemical properties by using cation size as a tuning parameter^7–10^. The structural stability and phase transition as a function of cation size have been studied by many groups and is available in the literature^11–14^.

The crystal structure and phase transitions in these oxides at high pressures are investigated several times by several groups. It is found that, the large cation sized RES are stable at high pressures whereas the small cation sized RES go through a transition from C $\rightarrow \,$ B $\rightarrow$ A. On the other hand, the sesquioxides of medium cation size follows a direct C $\rightarrow$ A transition at high pressures. Though these transitions are well known, a systematic study revealing the cation size dependent structural phase transitions has been primarily investigated on (Eu_1−*x*_Ho_*x*_)_2_O_3_ solid solutions by our own group^8^. This study was carried out by keeping the aim to investigate the structural stability of RES as a function of both cation size and pressure by considering the solid solutions of RES having similar structure and small difference in their cation size. The high pressure structural stability of cubic phase of (Eu_1−*x*_Ho_*x*_)_2_O_3_ solid solutions has been thoroughly studied and a pressure concentration phase diagram has been established recently^8^. In fact, this is the first experimental phase diagram delineating the phase boundaries of the high pressure phases (monoclinic and hexagonal) of these oxides as a function of cation size. The solid solutions of RES La_2_O_3_ and Eu_2_O_3_ having dissimilar structure and significant difference in their cation size has been recently synthesized and a thorough structural analysis has been carried out^11^. In this case, the microstrain and substitutional disorder developed by the difference in the cation size drives the polymorphic phase transition from C $\rightarrow$ B $\rightarrow$ A. Moreover, the observed structural phase transitions are as similar to the transition sequence observed in the RES at high pressures. In the present work these solid solutions of RES, La_2_O_3_ and Eu_2_O_3_ having dissimilar crystal structure and significant difference in their cation size, have been studied at high pressures and an attempt is made to map their phase structure and the compression behavior as a function of cation size and pressure.

## Results

### Structural phase transitions in (Eu_**1−*x***_La_***x***_)_**2**_O_**3**_

**Figure 1:**
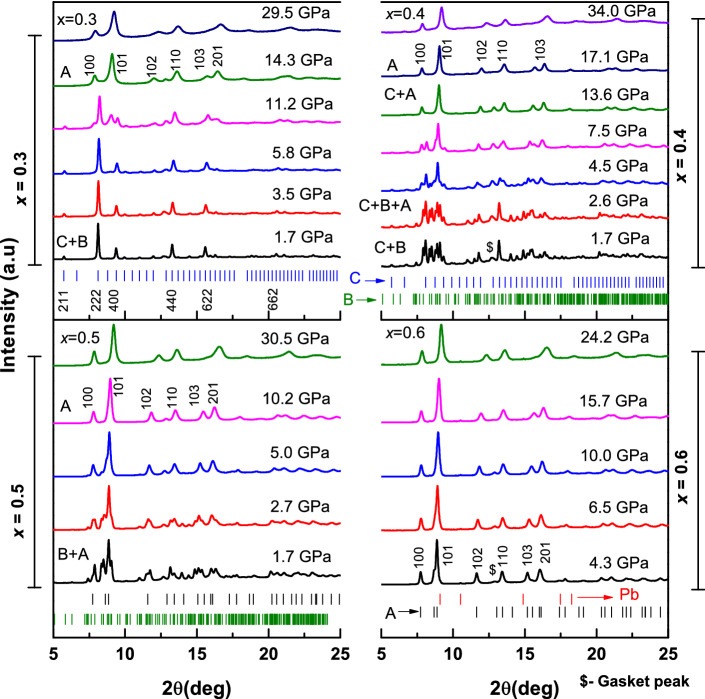
HPXRD pattern of *x* = 0.3–0.6 representing the high pressure behavior of (Eu_1−*x*_La_*x*_)_2_O_3_. C, B, A represents the cubic, monoclinic and hexagonal structures respectively. *x* = 0.6 (and *x* > 0.6) indicate the stability of hexagonal structure (A-type) up to the highest experimental pressure. ‘$’ symbol indicate the peak corresponding to the gasket material. A minor fraction of Lead (Pb) was observed due to the X-ray slit introduced to the DAC^19^.

A detailed description of synthesis of (Eu_1−*x*_La_*x*_)_2_O_3_ solid solution and their structural and morphological characterization are reported in our earlier study^11^. At ambient temperature and pressure(ATP), the crystal structure of rare earth sesquioxides is mainly dictated by their cation size. Due to the systematic variations of cation size, we see a C $\rightarrow$ B $\rightarrow$ A transition in this solid solutions with an increasing La content^11^. The solid solutions with *x* $<\,0.2$ crystallises in the pure cubic phase whereas a pure hexagonal phase is obtained for *x* $>\,0.6$. The solid solutions with 0.2 $\le$ *x* $\le$ 0.6 shows a multiphase character as they represents a progressive nature of C $\rightarrow$ B $\rightarrow$ A transition as reported in our previous study^11^. High pressure X-ray diffraction measurements have been carried out at various pressures on selected La compositions namely, $\textit{x} =0$, 0.2, 0.3, 0.4, 0.5, 0.6, and 0.8. Figure 1 shows the X-ray diffractograms recorded for $\textit{x}= 0.3$, 0.4, 0.5 and 0.6 at various pressures representing the behavior of (Eu_1−*x*_La_*x*_)_2_O_3_ solid solutions at high pressures. In our earlier study, pure La_2_O_3_ sample has been extensively investigated at high pressures and it has been reported to be stable in its ambient hexagonal structure^15^. It is seen that, the diffraction data for $\textit{x} = 0.8$ and 0.6 that is solid solutions with higher La content do not show any signature of structural phase transition till the highest experimental pressure, 27.4 and 24.2 GPa respectively. This indicates the stability of the hexagonal structure of these oxides at least up to 25 GPa similar to pure La_2_O_3_^15^. In the case of $\textit{x}= 0.6$, though the ambient data showed the presence of a small fraction of monoclinic phase along with the hexagonal phase^11^, the diffraction pattern at 4.3 GPa clearly showed that, the monoclinic phase is absent in this oxide at this pressure. This signifies that, the monoclinic phase is completely transformed to the hexagonal structure well below this pressure. Also, it has been observed that, the *100* peak of the hexagonal structure of $\textit{x}=0.8$ and 0.6 hardly shift with an increase of pressure in the ~ 10–22 GPa pressure regime, indicative of the presence of anomalous lattice compressibility, similar to the one in the hexagonal structure of pristine La_2_O_3_, Nd_2_O_3_ and Eu_2_O_3_^15,17,18^.

**Figure 2:**
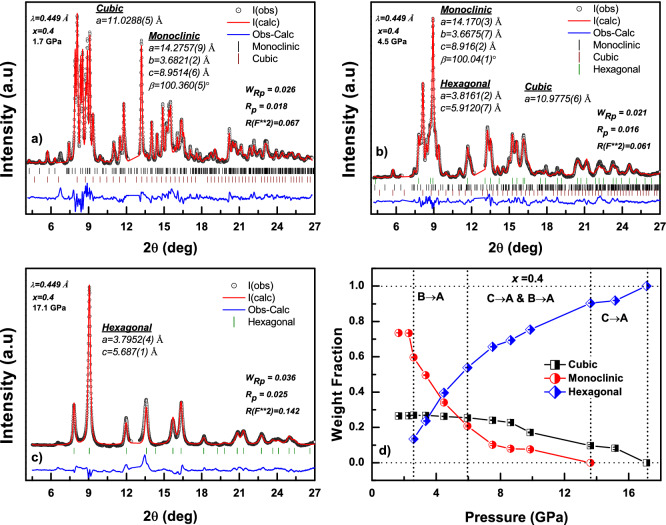
The representatives of Rietveld fit for *x* = 0.4 at various pressure steps; (**a**) at 1.7 GPa, (**b**) at 4.5 GPa and (**c**) at 17.1 GPa. The refined value of lattice parameters and the agreement indices are indicated in the respective plots. (**d**) The weight fraction of cubic monoclinic and hexagonal structures with increasing pressure showing the phase progression. The vertical dotted lines separate the different regions of $\hbox {B}\rightarrow \hbox {A}$ and $\hbox {C}\rightarrow \hbox {A}$ transitions.

For $\textit{x}=0.5$, a mixture of monoclinic and hexagonal phase is observed at ambient temperature and pressure (ATP)^11^. With an increase of pressure, the monoclinic phase was found to be gradually transforming to the A-type structure. This transition was completed at 10.2 GPa, where a pure A-type structure was observed. Further increase in pressure does not induce any structural phase transition and the A-type structure was found to be stable up to 30.5 GPa. In the case of $\textit{x}=0.4$, coexistence of cubic (type-C) and monoclinic (type-B) phase was observed and their presence have been verified using the Rietveld structure refinement of the ambient diffraction pattern^11^. As can be seen from the Fig. 1, the C + B mixed phase in the $\textit{x}=0.4$ composition completely transformed to the A-type structure at 17.1 GPa. Further increase in pressure up to 34 GPa does not show any structural phase transition in this oxide. However, it was difficult to say whether the $\hbox {C}\rightarrow \hbox {A}$ transition took place first or the $\hbox {B}\rightarrow \hbox {A}$. To remove this ambiguity, quantitative multi phase analysis using the Rietveld structure refinement have been carried out for all the High Pressure X-ray Diffraction (HPXRD) patterns. The representatives of Rietveld fit corresponding to $\textit{x}=0.4$ at various pressure steps, 1.7 GPa, 4.5 GPa and 17.1 GPa, are shown in Fig. 2a–c. The unit cell parameters for the cubic, monoclinic and the hexagonal structures along with the different agreement indices (R factors) are indicated in the respective figures. The low value of different agreement indices indicate the good quality of the Rietveld fit to the observed diffractogram. It is worth mentioning here that, the observed diffraction data at 2.6 GPa showed an appreciable intensity difference from the calculated one in the 2$\theta$ range 7.75°–8.00° when fitted with cubic and monoclinic phase alone. This suggests the possibility of the onset of hexagonal structure in this oxide at this pressure. Hence, the refinement has been carried out by including the hexagonal structure, and the difference in intensity was observed to be minimal. Thus we conclude that, the onset of C/B $\rightarrow$ A structural phase transition in this oxide is at 2.6 GPa. Further refinement has been carried out using all the three phases and the phase progression was tracked by means of weight fraction of individual phases. Figure 2d represents the weight fraction of cubic monoclinic and hexagonal structures with the increase of applied pressure. It is clear that, below 2.6 GPa, the cubic and monoclinic phase retains its fraction. At 2.6 GPa, the fraction of monoclinic phase started decreasing whereas the cubic phase does not show any appreciable change. Correspondingly, the fraction of hexagonal phase has grown to 13% and a similar amount of reduction was observed for the monoclinic phase. This drop and rise in the individual fraction of monoclinic and hexagonal structure, respectively, clearly indicates the monoclinic to hexagonal transition at 2.6 GPa. The fraction of cubic phase continued to remain constant till 5.9 GPa, where a fraction of 10% drop was observed. Also the fraction of monoclinic phase was dropped to 20% at this pressure. These indicate that, B $\rightarrow$ A is the dominant transition between 2.6 and 5.9 GPa. Above 5.9 GPa, a gradual reduction in the fraction of cubic and monoclinic phase was observed and B $\rightarrow$ A transition was complete below 13.6 GPa. This points to the fact that, between 5.9 and 13.6 GPa, both cubic and monoclinic phase contributes towards the growth of hexagonal structure. With further increase in pressure to 17.1 GPa, the remaining cubic phase transforms completely to the hexagonal structure. Beyond this pressure, the hexagonal structure was found to be stable up to 34 GPa.

In the case of $\textit{x}= 0.3$ and 0.2, a coexistence of cubic and monoclinic structure was observed at ATP^11^. However, the phase fraction of the monoclinic phase was significantly low in these two compositions^11^. Hence tracking the monoclinic phase with pressure was found to be extremely difficult. Moreover, considering the absorption of X-rays by diamonds in a DAC, the scattering from a phase with such a low fraction could not be well resolved. Hence, for $\textit{x}=0.3$ and 0.2, the analysis was carried out by focusing on the major cubic phase and the monoclinic phase was not considered further in the discussion. In Fig. 1 the HPXRD patterns collected for $\textit{x}= 0.3$ at different pressure steps are shown. Typically, the structural transitions either from C $\rightarrow$ B or C $\rightarrow$ A is characterized by the origin of a new peak near the cubic *123* and *222* peak^8^. For $\textit{x}=0.3$, the diffraction pattern doesn’t show any signature of structural phase transition till 5.8 GPa. The origin of a new peak at 8.96^0^ near the cubic *123* peak at 5.8 GPa indicate the onset of structural phase transition in this oxide. With further increase in pressure, the high pressure phase started growing and the transition was complete at 14.3 GPa. The high pressure phase could be indexed to the known hexagonal structure with space group *P*$\bar{3}$*m*1. This C $\rightarrow$ A transition was much similar to the one observed in the intermediate cation sized rare earth sesquioxides like Sm_2_O_3_, Eu_2_O_3_ and Gd_2_O_3_^8,20,21^. There was no structural phase transitions observed with further increase of pressure up to 29.5 GPa, indicating the stability of the hexagonal structure up to this pressure. For $\textit{x}=0.2$, a similar transition was observed at 4.3 GPa with the origin of a new peak at $2\theta = 9.02^{0}$. Since the transition pressure is comparatively lower than that of the $\textit{x}=0.3$, it is speculated that, the B $\rightarrow$ A transition could have taken place prior to the C $\rightarrow$ A transition as in the case of $\textit{x}=0.4$. However, it was difficult to confirm due to the low phase fraction and the absence of well resolved monoclinic peaks in the HPXRD data. The parent phase completely transformed to the high pressure phase below 14.1 GPa. The high pressure phase could be indexed to the hexagonal structure as in the case of $\textit{x}=0.3$. Further increase in pressure to 31.1 GPa, does not induce any structural phase transitions. Though the reports on the high pressure behavior of pure Eu_2_O_3_, $\textit{x}=0$, are available elsewhere in the literature^8^, the measurements has been repeated here to present an overall picture of the phase transitions. A phase transition from cubic to hexagonal structure is observed at 5.3 GPa and the high pressure phase is stable up to 28.1 GPa. These results are in good agreement with the available reports on Eu_2_O_3_^8^.

It is known that, the structure and phase transitions in the RES are highly influenced by their cationic radii^6,8,11,14,22^. It is reported that, an increase in the cationic radii decreases the transition pressure and bulk moduli and drives the phase transition route from C $\rightarrow$ B to C $\rightarrow$ A^8^. Also it has been shown that, these transitions from C $\rightarrow$ B $\rightarrow$ A can be realized by fine tuning the average cationic radii, R_RE_, in a systematic way^7,11,23^. The present high pressure studies on the (Eu_1−*x*_La_*x*_)_2_O_3_ solid solutions have now shown that, pressure also favours the same transition routes which are observed by increasing the R_RE_ of the solid solution. The deviation from this can be seen in the cubic systems, (for $\textit{x}=0$, 0.2, and 0.3), in which the increase in R_RE_ induces a C $\rightarrow$ B transition whereas pressure induces a direct C $\rightarrow$ A transition. This is due to the comparatively lower stability of the monoclinic phase of a particular composition (R_RE_ $\le$ 1.01 Å in this case) over the cubic phase when treated under pressure, as we have seen in the case of $\textit{x}=0.4$. It seems that, pressure drives the mixed phase, either C + B or B + A, towards the hexagonal structure which is the stable structure exhibited by the RES with large cationic radii at ATP. This indicate that, the C $\rightarrow$ B $\rightarrow$ A transition in (Eu_1−*x*_La_*x*_)_2_O_3_ observed with an increase in the R_RE_ is further enhanced with the increase of pressure.

### Lattice compressibility and equation of state

**Figure 3:**
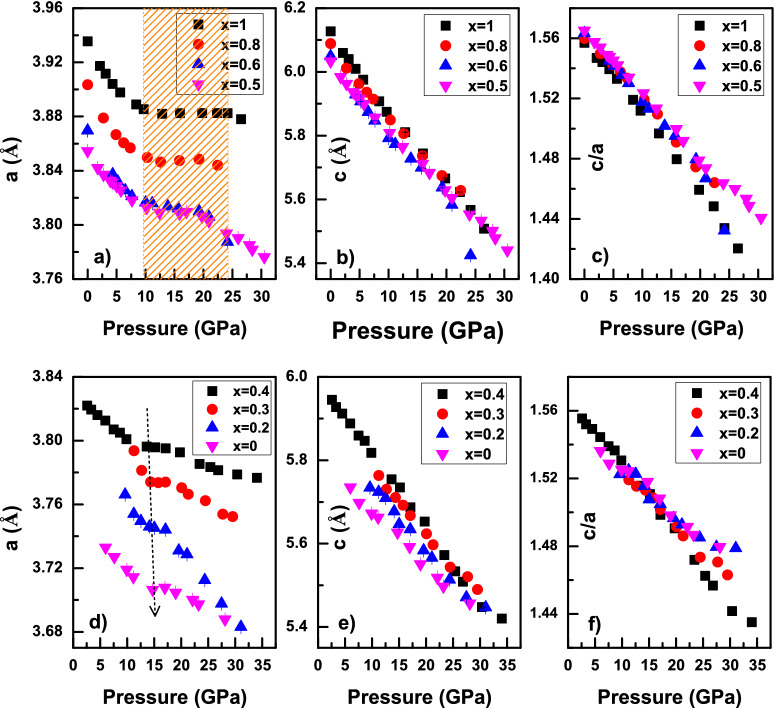
The variation of unit cell parameters and c/a ratio of hexagonal structure with increasing pressure. (**a**–**c**) for those compositions in which the hexagonal structure is stable at ATP, (**d**–**f**) for those compositions in which the hexagonal structure is stable at high pressure. The shaded region represents the pressure width where the anomaly is visible for the pure La_2_O_3_. The downwards dotted arrow represents the shift in onset of the anomalous compression in the high pressure phase.

Figure 3a–c shows the change in lattice parameters with increasing pressure for those compositions in which the hexagonal structure is found to be stable at ATP. It is clear that, the anomalous lattice compressibility along the direction of *a* axis is present in all the hexagonal structures irrespective of the compositions studied. This is due to the shifting of cations from the *103* plane to the *100* plane as a consequence of the pressure induced layer sliding as indicated for the hexagonal La_2_O_3_^15^. The pressure width of the anomalous region for the hexagonal phase of pure La_2_O_3_ is about 9.7–24.2 GPa, whereas the region is shifted to 12.4–19.8 GPa in the case of $\textit{x}=0.5$. This implies that, not only the onset of the anomaly is shifted to a high pressure region but also, the pressure width where the anomaly is observed shifts to a lower value with a decrease in R_RE_. This could point to the fact that, the RE-O layer sliding is getting restricted with decrease of R_RE_ and becomes insignificant at sufficiently low value of R_RE_. Though there is an anomaly along the *a* axis, typical decreasing trend is seen along the *c* axis of these compositions. Moreover, the *c/a* ratio shown in Fig. 3c does not show any sudden jump rather it shows a gradual slope change with increase of pressure. Figure 3d–f shows the change in lattice parameters with increasing pressure for those compositions in which the hexagonal structure is found to be stable at high pressures. In this case, the anomaly still persists and it is found to be shifting towards the high pressure region with decreasing R_RE_. The compression along the *c* axis and the *c*/*a* ratio follow a similar trend as earlier. This atypical behavior in the hexagonal structure of all the solid solutions leads to the conclusion that the anomalous lattice compression is an inherent property of the hexagonal structure of rare earth sesquioxides.

To investigate the unit cell compressibility behavior, the pressure–volume (P–V) data of parent and high pressure phases has been fitted to the known third order Birch–Murnaghan equation of state (BM-eos) of the form,

1 $$\begin{aligned} P(V)=\frac{3}{2}B_{0}\left[ \left( \frac{V_{0}}{V}\right) ^{\frac{7}{3}}-\left( \frac{V_{0}}{V}\right) ^{\frac{5}{3}}\right] \left\{ 1+\frac{3}{4}\left( B_{0}^{'}-4\right) \left[ \left( \frac{V_{0}}{V}\right) ^{\frac{2}{3}}-1\right] \right\} \end{aligned}$$

**Figure 4:**
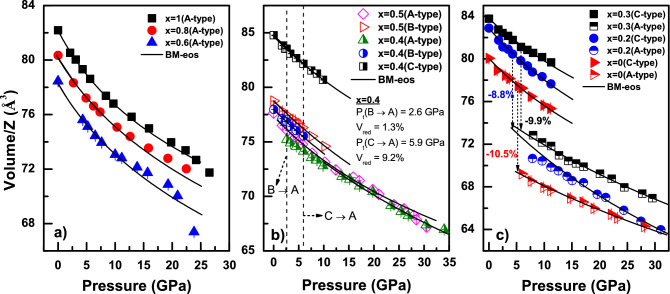
The experimental pressure volume (P–V) data for all the investigated oxides. The solid lines indicate the Birch–Murnaghan equation of state fit to the P–V data. The fitting parameters and volume drops at the transition pressure, P_t_, are tabulated in Table 1.

**Figure 5:**
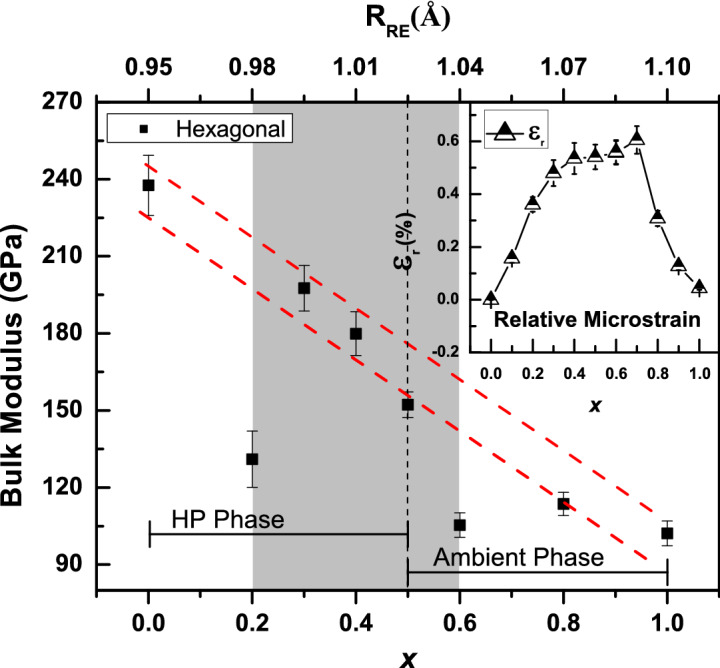
The variation of Bulk modulus of the hexagonal structure with *x*/R_RE_. The red dotted lines are guide to the eye, showing the decreasing trend of bulk modulus with increase of R_RE_. The vertical dotted line at $\textit{x}=0.5$ separate the hexagonal phase at ATP from the one at high pressure. The variation in relative micro strain reproduced from our earlier reports on (Eu_1−*x*_La_*x*_)_2_O_3_ solid solutions is shown in the inset^11^.

Here $B_{0},B_{0}^{'},V_{0}$ and *V* are the bulk modulus, derivative of bulk modulus, volume at ambient pressure and volume at pressure *P* respectively. The bulk moduli of the parent and the high pressure structures of all these oxides were obtained by fitting the experimental data to the above eos. Only the P-V data below 10 GPa is considered for eos fitting to the parent phase whereas the data above 15 GPa is used for the high pressure phases. A value of $B_{0}^{'}=9.8$ was obtained by fitting the compressibility data of pure La_2_O_3_, $\textit{x}=1$, in our earlier study^15^. This value has been used (fixed) to fit the compressibility data of hexagonal structures of those compositions for which the hexagonal structure was stable at ATP. In the case of cubic phase of $\textit{x}=0$, the data is fitted to the BM-eos and a value of $B_{0}^{'}= 7.4$ is obtained. This value has been used (fixed) in fitting the compressibility data of all the cubic phases. These values were adopted to provide a better comparison with the end members. All other BM-eos fitting were carried out with a fixed value of $B_{0}^{'}=4$. In Fig. 4a–c, the BM-eos fit to the pressure dependence of the unit cell volume for the different phases of all the compositions are shown. The bulk modulus values derived from the eos fittings are tabulated in Table 1. It can be seen that, the bulk modulus of cubic structure is increased from 142(5) to 167 (2) GPa when the composition is changed from $\textit{x}=0$ to 0.4. For $\textit{x}=0.4$, the bulk modulus values of monoclinic and the high pressure hexagonal structure are 168(3) GPa and 180(8) GPa respectively, which are in close agreement with the values obtained for the cubic structure of the same composition, ie, 167(2) GPa. This indicates that, at $\textit{x}=0.4$, the cubic, monoclinic and hexagonal structures show a similar compressibility behavior. Further, the variation in the bulk modulus of the hexagonal structure with composition, *x*, and cation size, R_RE_, is shown in the Fig. 5. A monotonous decrease in the bulk modulus can be seen with the increasing *x*/R_RE_ except for $\textit{x}=0.2$ and 0.6. This decreasing trend of bulk modulus is again attributed to the decreasing bond strength with an increase of *x*/R_RE_ as observed in the case of (Eu_1−*x*_Ho_*x*_)_2_O_3_ system^8,14,24^. Whereas, the reduction in bulk modulus for $\textit{x}=0.2$ and 0.6 is due to the presence of maximum micro strain in these compositions^11^. For convenience, the variation of the micro strain, $\varepsilon _{r}$, in the parent structures relative to the end member as a function of increasing *x*/R_RE_ is reproduced from the literature^11^ and shown as the inset of Fig. 5. Though the micro strain is present in all the solid solutions, it is yet not significant to alter the bulk modulus values considerably in compositions with low $\Delta$R_RE_(difference in cation size). It is evident that the strain is maximum for the compositions with $\textit{x}=0.2$ and 0.6. This increased micro strain manifests as an internal pressure in the crystal structure leading to the reduction in the structural stability and hence a reduction in bulk modulus for $\textit{x}=0.2$ and 0.6. Similar behavior was seen in our earlier investigation of (Eu_1−*x*_Ho_*x*_)_2_O_3_ system and also in several other mixed RES systems^8,25,26^.

**Table 1:** Bulk moduli, transition pressure and volume reduction for all the mixed oxides (Eu_1−*x*_La_*x*_)_2_O_3_ compositions along with the reported values of the end members.

*x*	B_0C_ (GPa)	B_0B_ (GPa)	B_0A_ (GPa)	P_t_ (GPa)	V_red_ (%)
0	142(5)		238(12)	5.3	10.5
	140(3)^8^		155(10)^8^	5.8^8^	10.3^8^
0.2	133(3)		131(11)	4.3	8.8
0.3	173(4)		198(9)	5.8	9.9
0.4	167(2)	168(3)	180(8)	2.6/5.9	1.3/9.2
0.5		169(2)	152(5)		
0.6			105(5)		
0.8			113(5)		
1			102(5)^15^		
			113(1)^16^		

Transition pressure, P_t_, and volume reduction, V_red_, from $\hbox {B}\rightarrow \hbox {A}$ is separated by a /(slash) in respective columns.

### Phase diagram of (Eu_1−*x*_La_*x*_)_2_O_3_

**Figure 6:**
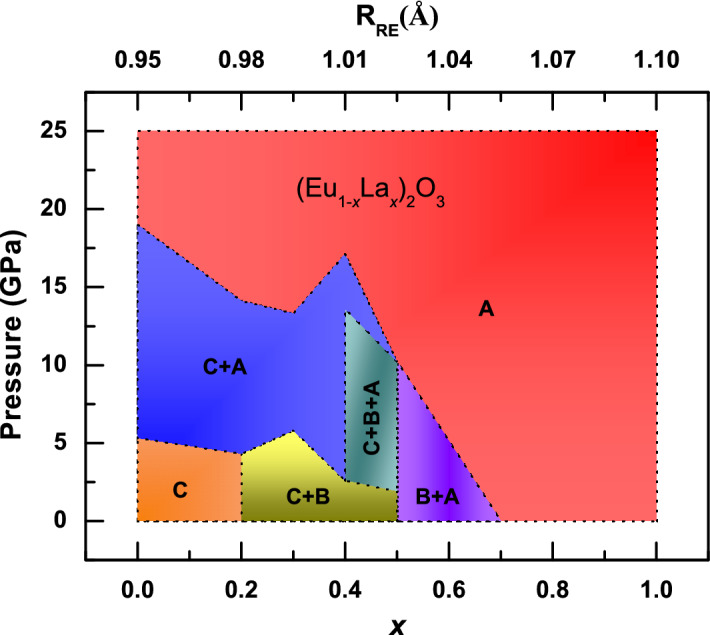
Pressure–concentration (P–*x*) phase diagram for the solid solution (Eu_1−*x*_La_*x*_)_2_O_3_. The phase labels C, B, A are used to represent cubic, monoclinic and hexagonal structures respectively. Single, di and tri phasic regions of different crystal structures and their stability regions are marked accordingly.

A pressure–concentration (P–*x*) phase diagram upto a pressure of 25 GPa is established from our HPXRD investigation on (Eu_1−*x*_La_*x*_)_2_O_3_ and is shown in Fig. 6. The stability region of the different polymorphic structures are marked with C, B and A respectively for cubic, monoclinic and hexagonal. The bi/triphasic regions were marked as a combination of these. Average cationic radii corresponding to each *x* is calculated using the definition of R_RE_ described earlier^14^. The cubic structure stable for 0.95 Å $\le$ R_RE_ $<\,0.98$ Å at ambient temperature and pressure prefers C $\rightarrow$ A transition with increasing pressure. The biphasic region of cubic and monoclinic structure stable for 0.98 Å $\le$ R_RE_$<\,1.025$ Å at ambient temperature and pressure prefers C/B $\rightarrow$ A transition at high pressures. Further, in the biphasic region of monoclinic and hexagonal structure favoured for 1.025 Å $\le$ R_RE_ $<\,1.055$ Å, the B-type structure progress towards the hexagonal A-type under pressure. The pure A phase obtained for 1.055 Å $\le$ R_RE_ $\le$ 1.10 Å was structurally stable at high pressures.

## Materials and methods

The solid solutions of Eu_2_O_3_ and La_2_O_3_ were synthesized using a simple soft chemistry approach^11,14^. Since, the synthesis procedure is available in our earlier report, only a brief description is given here. C-type Eu_2_O_3_ and A-type La_2_O_3_ (99.9% purity, Alfa Aesar) were weighed according to the nominal formulae, (Eu_1−*x*_La_*x*_)_2_O_3_, and dissolved in dilute nitric acid(1:3). The nitrate solution was then heated at 200 °C on a hot plate to remove the excess nitric acid. After cooling down, it was mixed with citric acid and heated (200 °C) again on a hot plate until a viscous gel was obtained. The gel was decomposed in air at high temperatures and calcined at 400 °C to remove the excess carbon present in it. The powder thus obtained was ground well and heat treated at 900 °C for 12 hours. The detailed description regarding the synthesis method is reported in our previous study^11^. The results of the detailed structural characterization of these solid solutions at ATP using the Rietveld structure refinement have been reported in our earlier study^11^. The study showed that the crystallites are irregularly shaped with a submicron size. The same samples were used in the present high pressure studies. A Mao-Bell type Diamond Anvil Cell (DAC) was used to generate the high pressures^19^. The powder sample, pressure calibrant and the pressure transmitting medium were loaded in to the 250 *µm* hole drilled at the center of a preindented steel gasket. The R_1_ fluorescent line shift of the ruby crystal was used to calibrate the pressure inside the hole. Silicone oil was used as the pressure transmitting medium in all the studies. Angle dispersive high pressure X-ray diffraction measurements (HPXRD) were carried out in transmission geometry at BL-11 of Indus-2 synchrotron facility at Raja Ramanna Centre for Advanced Technology (RRCAT), Indore, India. A Si(111) channel cut monochromator was used to monochromatise the X-ray beam and an X-ray wavelength of $\lambda =0.449\mathring{A}$ was selected for the present study. The diffracted X-rays were collected using the *mar345* image plate detector. Fit2D programme was used to convert the 2D powder diffraction data in to the one dimensional $2\theta$ versus intensity^27^. Rietveld refinement of the high pressure data was carried out using the GSAS + EXPGUI software package^28,29^. The crystal structure of La_2_O_3_ and Eu_2_O_3_ obtained from our previous studies was used as the model structure^11,17^.

## Supplementary information

Supplementary file1 (pdf 1259 KB)
