# Supplementary material for: Structural phase transition, equation of state and phase diagram of functional rare earth sesquioxide ceramics (Eu1−xLax)2O3
Source: Sci Rep. 2020 Jul 16;10:11829. doi: 10.1038/s41598-020-68400-9 (PMC7366723; doi:10.1038/s41598-020-68400-9)
Supplement: Supplementary file 1 — Supplementary file1 (pdf 1259 KB) [file 41598_2020_68400_MOESM1_ESM.pdf]

Structural phase transition, equation of state and phase diagram of functional rare earth sesquioxide ceramics $(Eu_{1-x}La_x)_2O_3$

K. A. Irshad[1,*], V. Srihari[2], S. Kalavathi[1], and N.V. Chandra Shekar[1]

[1]High Pressure Physics Section, Condensed Matter Physics Division, Materials Science Group, HBNI, Indira Gandhi Centre for Atomic Research, Kalpakkam 603102, India
[2]High Pressure & Synchrotron Radiation Physics Division, Bhabha Atomic Research Centre, 400085, Mumbai, India
[*]irshad@aigcar.gov.in

ABSTRACT

The intriguing functional nature of ceramics containing rare earth sesquioxide (RES) is associated with the type of polymorphic structure they crystallize into. They prefer to be in the cubic, monoclinic or hexagonal structure in the increasing order of cation size, R_{RE}. Since the functional properties of these ceramics varies with R_{RE}, temperature and pressure, a systematic invesigation delineating the cation size effect is indispensable. In the present work we report the structural stability and compressibility behaviour of the RES ceramics, $(Eu_{1-x}La_x)_2O_3$, of RESs with dissimilar structure and significant difference in cationic radii. The selected compositions of $(Eu_{1-x}La_x)_2O_3$ have been studied using the *in-situ* high pressure synchrotron X-ray diffraction and the structural parameters obtained through Rietveld refinement. The cubic structure, which is stable for 0.95 Å $\leq R_{RE} <$ 0.98 Å at ambient temperature and pressure (ATP), prefers a cubic to hexagonal transition at high pressures. The biphasic region of cubic and monoclinic structure, which is stable for 0.98 Å $\leq R_{RE} <$ 1.025 Å at ATP, prefers a cubic/monoclinic to hexagonal transition at high pressures. Further, in the biphasic region of monoclinic and hexagonal structure, observed for 1.025 Å $\leq R_{RE} <$1.055 Å, the monoclinic phase is found to be progressing towards the hexagonal phase with increasing pressure. The pure hexagonal phase obtained for 1.055 Å $\leq R_{RE} \leq$ 1.10 Å is found to be structurally stable at high pressures. The bulk moduli are obtained from the Birch-Murnaghan equation of state fit to the compressibility data and its dependance on the cation size is discussed. The microstrain induced by the difference in cation size causes an internal pressure in the crystal structure leading to a reduction in the bulk modulus of x=0.2 and 0.6. A pressure-concentration (P-x) phase diagram upto a pressure of 25 GPa is constructed for $(Eu_{1-x}La_x)_2O_3$. This would provide an insight to the fundamental and technological aspects of these materials and the RESs in general.

Supplimentary figures

The X-ray diffraction pattern of selected compositions of $(Eu_{1-x}La_x)_2O_3$collected at high pressures are shown bellow.

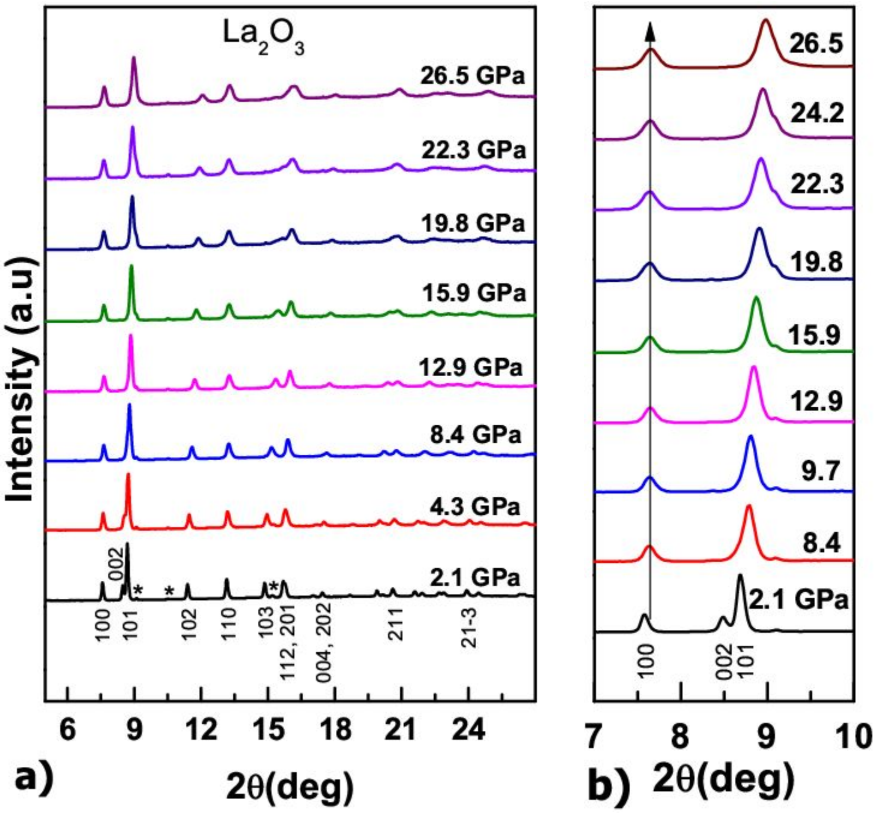

Figure 1. a)High Pressure X-ray Diffraction(HPXRD) pattern of pure hexagonal La_2O_3 (space group $P\bar{3}m1$) collected at various pressure steps. * marks indicate the Lead peaks observed due to the X-ray slit near the diamond table[1] b) The shift of *100* peak of the hexagonal structure with pressure. It can be seen that the *100* peak is hardly moving with pressure in the 9.7-22.3 GPa pressure region. The upward arrow is guide to the eye. The figure is taken from the reference[2]

References

1. Irshad, K. A., Sanjay Kumar, N. R. & Chandra Shekar, N. V. A novel and simple x-ray slit for diamond anvil cell based x-ray diffraction experiments. *Meas. Sci. Technol.* **28**, 047002, DOI: 10.1088/1361-6501/aa5e25 (2017).
2. Irshad, K. A. *et al.* Anomalous Lattice Compression in the Hexagonal La_2O_3 - A high pressure X-ray diffraction, Raman spectroscopy and First principle study. *J. Alloy. Compd.* **822**, 153657, DOI: 10.1016/j.jallcom.2020.153657 (2020).

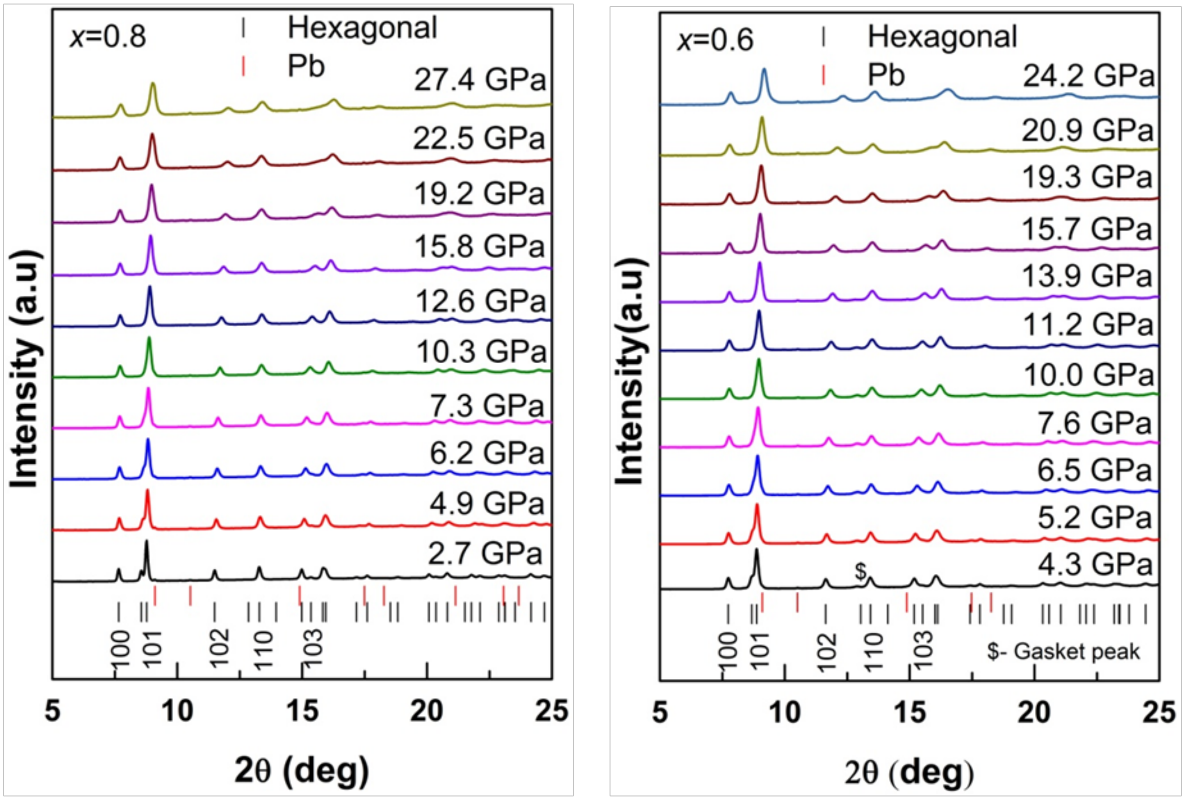

Figure 2. HPXRD pattern of $x = 0.8$ and 0.6 at various pressures showing the stability of hexagonal structure up to the highest experimental pressure. '$' symbol indicate the peak corresponding to the gasket material. A minor fraction of Lead (Pb) was observed due to the X-ray slit introduced to the DAC[1].

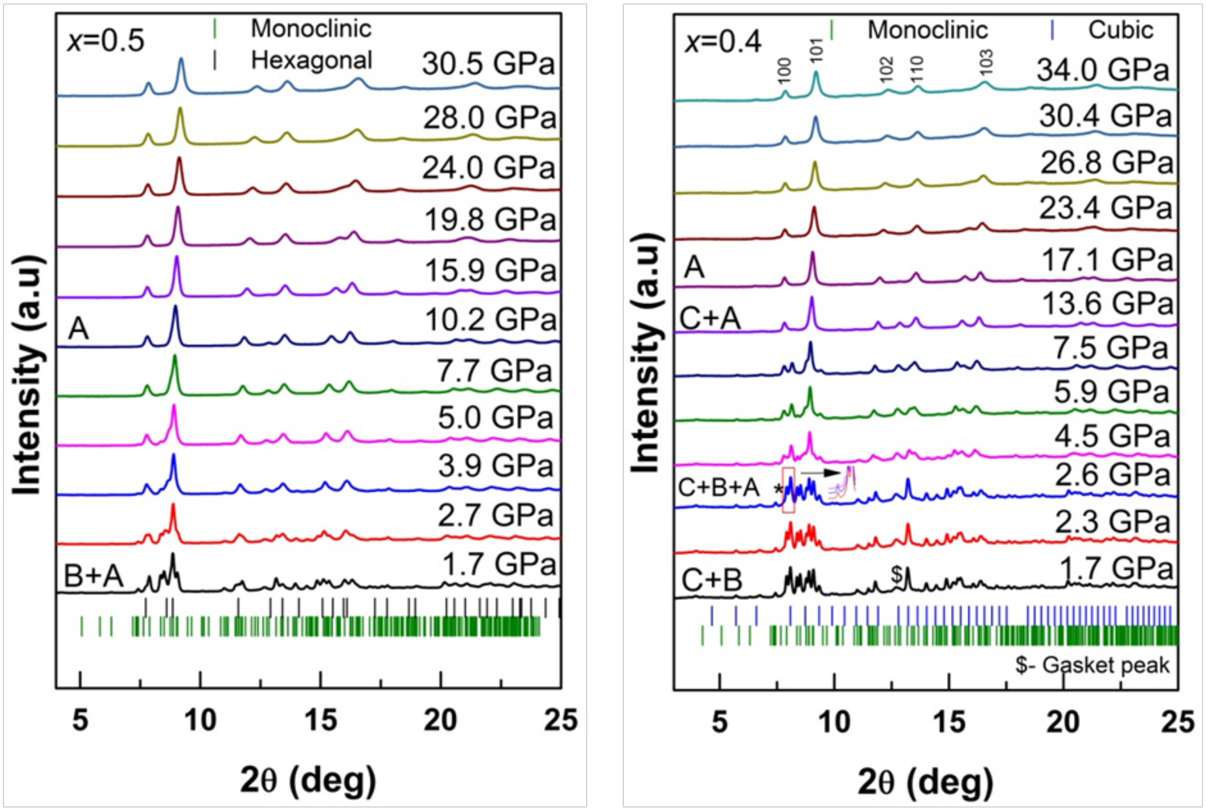

Figure 3. HPXRD pattern of $x = 0.5$ and 0.4 at various pressures. C, B, A represents the cubic, monoclinic and hexagonal structures respectively. '$' symbol indicate the peak corresponding to the gasket material. The tick marks represent the reflections belonging to the corresponding phases.

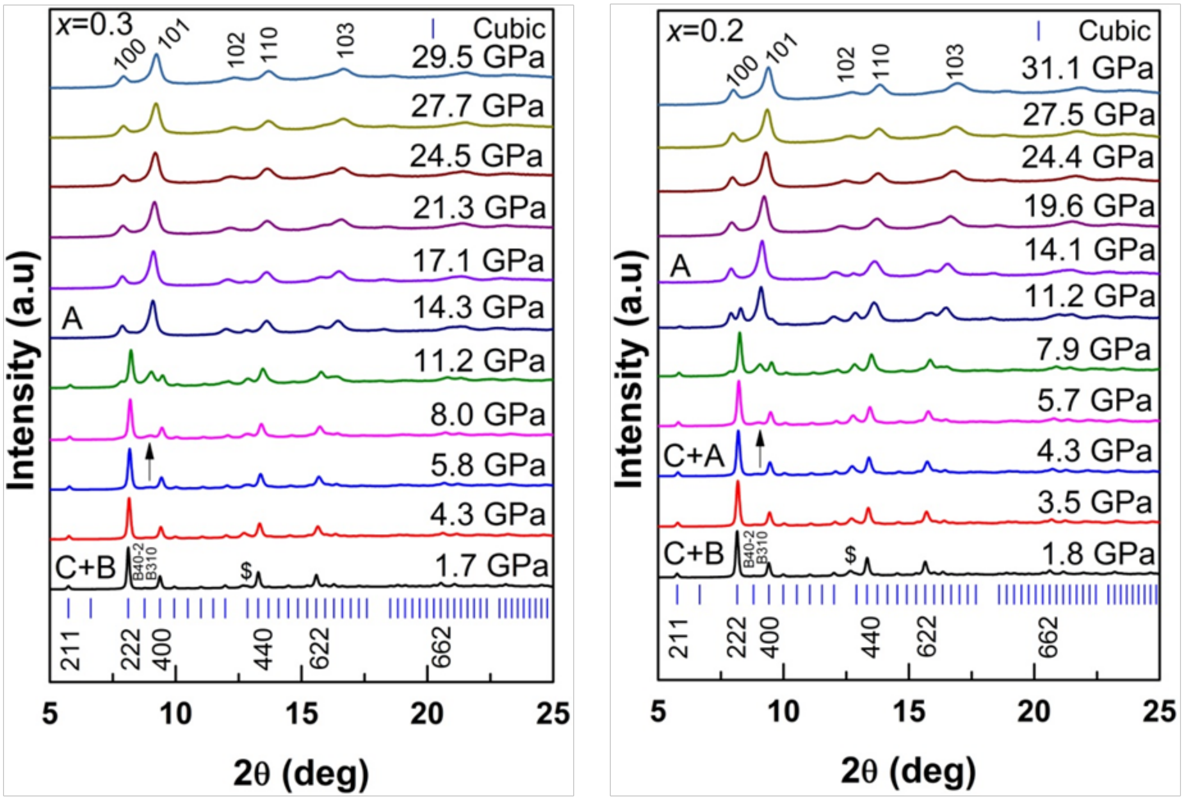

Figure 4. HPXRD pattern of $x = 0.3$ and 0.2 at various pressures. The onset of C/B$\rightarrow$ A phase transitions is indicated by up arrows. C, B, A represents the cubic, monoclinic and hexagonal structures respectively. "$" symbol indicates the peak corresponding to the gasket material. The tick marks represent the reflection positions belonging to the individual phases.
